# Rural-Urban Differences in Maternal Responses to Childhood Fever in South East Nigeria

**DOI:** 10.1371/journal.pone.0001788

**Published:** 2008-03-12

**Authors:** Benjamin S. C. Uzochukwu, Emmanuel O. Onwujekwe, Chima A. Onoka, Maduka D. Ughasoro

**Affiliations:** 1 Department of Community Medicine, College of Medicine, University of Nigeria, Enugu, Nigeria; 2 Department of Health Administration and Management, College of Medicine University of Nigeria, Enugu, Nigeria; 3 Department of Community Medicine, University of Nigeria Teaching Hospital, Enugu, Nigeria; 4 Department of Paediatrics, University of Nigeria Teaching Hospital, Enugu, Nigeria; Swiss Tropical Institute, Switzerland

## Abstract

**Background:**

Childhood fevers due to malaria remain a major cause of morbidity and mortality among under-five children in Nigeria. The degree of vulnerability perceived by mothers will affect their perception of the severity and threat of their child's fever and the patterns of health care use. This study was undertaken to compare maternal responses to childhood fever in urban and rural areas of Enugu, south east Nigeria.

**Methodology/Principal Findings:**

Data was collected with pre-tested interviewer-administered questionnaires from 276 and 124 urban and rural households respectively. In each household, only one woman aged 15–49 years who had lived in each of the urban and rural communities for at least one year and had at least one child less than 5 years old was interviewed. Malaria was mentioned as the commonest cause of childhood fevers. Rural mothers were more likely to recognize danger signs and symptoms than urban mothers. Rural mothers use more of informal than formal health services, and there is more home management of the fever with urban than rural mothers. Chloroquine, ACT, SP and Paracetamol are the main drugs given at home for childhood fevers, but the rural mothers were more likely to use leftover drugs from previous treatment to treat the fevers than urban mothers. The urban respondents were also more likely to use a preventive measure. Urban mothers sought actions faster than rural mothers and the total cost of treatment was also higher in urban areas.

**Conclusions/Significance:**

Both urban and rural mothers are aware that malaria is the major cause of childhood fevers. Although rural mothers recognize childhood fever and danger signs better than urban mothers, the urban mothers' responses to fever seem to be better than that for rural mothers. These responses and differences may be important for geographical targeting by policy makers for malaria interventions.

## Introduction

Fever is a very common symptom in childhood illnesses with malaria accounting for most of the childhood fever in our environment [1], [2]. In Nigeria, childhood fevers presumed to be malaria remains a major cause of morbidity and mortality among under-five children accounting for 30% of all childhood deaths, 11% of maternal deaths and 50% of all out patient department attendance [3]. Also, about 50% of the population has at least one episode of malaria each year with children under the age of 5 years having 2 to 4 attacks of malaria a year [3]. All these have been attributed to ignorance and poor service delivery [4], [5]. Although, treatment often begins early and at home, a mother's inability to correctly recognize malaria has contributed substantially to child morbidity and mortality due to malaria [6]. In Nigeria, studies show that mothers were unable to recognize severe malaria despite perceiving the signs and symptoms of onset of childhood malaria as including high temperature and loss of appetite [7].

Parents especially mothers, most times get excessively concerned about their febrile child. Their misconception about fever, frequently prompt unnecessary contacts with the health care system and mishandling of fever generate a great deal of unwarranted parental anxiety resulting in avoidable medical complications, countless calls and costly visits to doctors' clinics and emergency rooms [8], [9] .

Health care in Nigeria currently concentrates on the rural population despite the rapid urbanization, rural-to-urban migrations and community degradation as in other sub Saharan Africa. The percentage of urban population in Nigeria is about 44.1% with an urban population annual growth rate of 5.5. The percentage of urban children is put at 32.3% [10] and the main health problems of these children in the urban populations are malaria, measles, tetanus, helminthic infestations, acute respiratory infections, diarrhea diseases and nutritional problems [11]. Although these health problems are common to both urban and rural environments, recognizing and meeting the public health challenges in these growing cities of Nigeria is becoming increasingly urgent as there are evidence that the urban poor are at far higher risk from malaria than previously acknowledged [12], [13].

There are several features that distinguish urban and rural malaria. Urban malaria occurs in a diverse and rapidly changing environment with high levels of human migration and high-density populations. Malaria transmission intensities in urban areas are different to those of rural areas. In urban areas, risk factors that may be different to those in rural areas lead to different disease burdens [14]. It has also been noted that rural and urban populations differ in several ways including their cultural practices, socioeconomic and demographic characteristics, availability and accessibility to formal and informal treatment sources, provision of basic infrastructure and childhood nutritional status [1], [15]–[17].

The degree of vulnerability perceived by mothers will affect their perception of the severity and threat of their child's fever and the patterns of health care use and over concern with body temperature termed “fever phobia” interferes with a mother's ability to accurately observe that her child, despite the fever, looks and acts normally [2], [8], [18]. Mother's quantification of fever serves to define the severity of the child's illness and is perceived as more meaningful to them than other symptoms that the child may be exhibiting [19]. In turn, this may prevent mothers from observing the potentially more serious signs and symptoms such as lethargy and dehydration, which warrant medical attention.

Proven effective options to reduce morbidity and mortality from fever/malaria include early diagnosis combined with prompt, effective therapy, and malaria prevention through the use of insecticide-treated nets (ITNs). Surveys in Africa revealed that 80–90% of fever presumed to be malaria cases were treated at home [20]–[22]. Frequently formal health care is sought only if initial treatment fails. Caregivers' behaviour in response to signs of disease is influenced by several factors including: knowledge, attitudes and practices towards malaria; accessibility and availability of health services; socio-economic factors, and perceptions of severity of the illness [2].

Several studies have compared maternal responses to childhood fevers in African rural and urban residents [1], [23]–[25]. In such studies, it was found that rural mothers use modern medical services and drugs as do urban mothers. However, individual, socio-cultural and structural factors influence the use of antimalarial drugs for episodes of fever presumed to be malaria. There is dearth of such studies in Nigeria. This study therefore set out to determine whether mothers perceive childhood fevers as a serious problem and to compare the knowledge, attitudes and treatment practices of urban and rural mothers with respect to childhood fevers. The result will facilitate geographical targeting by policy makers for malaria interventions.

## Methods

### Study area

This study was carried out in one urban (Uwani.) and one rural (Amechi-Awkunanaw) communities in Enugu south local government area of Enugu state, south east Nigeria from April to August 2005. While Uwani community is made up of 15 neighbourhoods, Amechi-Awkuananaw is made up of 6 villages. The 2 communities are about 15 kilometers apart and lie within the Guinea-Savanna forest belt with annual rainfall of 1520–2030 mm and the temperature ranges between 22.4 and 30.8 degree Celcius [26]. The vegetation is tropical rain forest with two major seasons, namely dry (November–April) and wet (May–October). The two communities have high malaria transmission rates year- round, with an average malaria incidence rate of 15% and 20% respectively. The major malaria vector in both communities is *Anopheles gambiae* while *Plasmodium falciparum* causes more than 90% of all malaria infections [27].

The 2006 projected population (from 1991 census) for Uwani is 49,133 while the population for Amechi-Awkunanaw is 20,498. A majority of both urban and rural residents are Ibos and Christians. They are mainly civil servants and traders in Uwani, and farmers and petty traders in Amechi-Awkunanaw. There are two primary health centres in Uwani and one in Amechi-Awkunanaw. This is complemented with many private clinics and patent medicine dealers (a person without formal pharmacy training, who sells orthodox pharmaceutical products on a retail basis for profit) [28] in Uwani and fewer of these and traditional healers in Amechi-Awkunanaw.

### Study design

The study was a descriptive cross-sectional study to determine whether mothers perceive childhood fevers as a serious problem and to compare the knowledge, attitudes and treatment practices of urban and rural mothers with respect to childhood fevers.. Enugu South Local Government was chosen for this study by simple random sampling from a frame of 4 local Government Areas (LGAs) with both urban and rural communities. Within Enugu South LGA, Uwani and Amechi-Awkunanaw communities were purposively chosen because they are typical urban and rural areas respectively in the local government area. In Uwani, 10 neighborhoods were chosen from a sample frame of 15 neighborhoods and in Amechi Awkunanaw, 3 villages were chosen from a sample frame of 6 villages. Adequate sample size was computed to be able to detect statistical significant differences between urban and rural respondents and also to ensure a high precision of collected data using a power of 80%, 95% confidence level and a knowledge of fever as a danger sign of 51% [29]. The EPI-info software version 6.04 [30] was used to calculate the sample size. The allocation of this sample size into those for urban and rural areas was done using proportionate allocation method considering their population resulting in a sample size of 280 households for Uwani and 130 households for Amechi-Awkunanaw. These households were therefore selected by simple random sampling from a sampling frame of the primary health care house numbering system.

### Data collection methods

Using pre-tested interviewer-administered questionnaires, information was collected from mothers aged 15–49 years who have lived in these areas for at least one year and have at least one child less than 5 years old. Only one woman per household was interviewed. The information collected included their socio-demographic characteristics, knowledge of fever and its occurrence in the past one month, perception of seriousness of childhood fever, recognition of signs and symptoms indicating deterioration of child's condition and help-seeking practices when a child has fever. For those who did not get better after their first action, where they sought help for the second action was determined. Only the actions taken for one child per household were recorded. Six interviewers with minimum of secondary school certificate and who were trained in interviewing techniques for 3 days conducted the interviews.

### Data analysis

The household data were checked daily for inaccuracies and inconsistencies by the authors, before double entry using the Epi-info version 6.04 [30]. Associations between urban and rural caregivers' characteristics and responses to fever were analysed using Pearsons' chi-square and Fisher's exact tests for categorical variables and Mann Whitney two-sample non-parametric test for continuous variables. A p-value of 0.05 was considered to be statistically significant.

### Ethical consideration

The study and verbal consent process received ethical approval from the Ethical Committee, University of Nigeria Teaching Hospital, Enugu. Verbal consent was collected from all the participating respondents. Written consent was not possible because experience has shown that in this environment, people are averse to signing signatures which they usually think might be used for Tax assessment purposes. The field workers went to the field with a register containing the household numbers and the participants who gave verbal consent were ticked and interviewed and those who refused were skipped. In addition, the authors verified this verbal consent process in the field. Participation was voluntary. Confidentiality of all information obtained from participants was maintained by not allowing information to be accessible to non-members of the research team.

## Results

### Socio-demograhpic characteristics of respondents

A total of 276 questionnaires were retrieved from 280 households in the urban area and 124 questionnaires from 130 households in the rural area. As shown in table 1, the mean ages of the urban and rural respondents are 31 (standard deviation (SD) = 6.9) and 36 (SD = 8.95) years respectively with a majority of the urban respondents falling within the age range of 26–30 years (34.8%) and rural. 36–40 years (25.8%). A greater percentage of the urban respondents (87.3%) had either secondary or tertiary education while only 6.9% had no formal education. However, a greater percentage of the rural respondents (62.1%) had either primary or secondary education. A good percentage of the rural respondents (27.4%) had no formal education. Most of the urban respondents are mainly traders (33.7%) and civil servants (30.0%), while professionals (1.4%) and farmers (0.4%) were the least among the respondents. On the other hand, 43.5% of rural respondents are traders and farmers (25.0%). There were very few professionals (0.8%) and unemployed (0.8%).

**Table 1:** Socio-demographic characteristics of respondents

Variables	Urban households	Rural households
	(n = 276)	(n = 124)
	N (%)	N (%)
**Age (years)**		
30 and less	152 (55.1)	37 (29.8)
>30	124 (44.9)	87 (70.2)
Mean (SD)	31 (6.9)	36 (8.95)
**Level of Education**		
No formal education	19 (6.9)	34 (27.4)
Primary school	16 (2.8)	38 (30.6)
Secondary school	128 (46.4)	39 (31.5)
Undergraduate	25 (5.4)	3 (2.4)
Tertiary education	98 (35.5)	10 (8.1)
**Occupation**		
Civil servant	83 (30,0)	12 (9.7)
Farmer	1 (0.4)	31 (25.0)
Business/trading	93 (33.7)	54 (43.5)
Artisans	22 (8.0)	9 (7.3)
Professionals	4 (1.4)	1 (0.8)
Students	26 (9.4)	6 (4.8)
Housewife	30 (10.9)	10 (8.1)
Unemployed	17 (6.2)	1 (0.8)

### Causes and danger signs and symptoms of childhood fever

Table 2 shows that the respondents in both rural and urban communities were able to identify one form or the other of causes of fever as well as danger signs of childhood fever. Malaria was mentioned as the commonest cause of childhood fevers with rural mothers being more likely to mention malaria than urban mothers (84% urban vs. 100% rural). Rural mothers were also more likely to recognize danger signs and symptoms that warrant medical attention than urban mothers like convulsion, strange breathing and anaemia. The differences between the urban and rural respondents were statistically significantly different as shown in table 2. None of the mothers was aware of all the causes and danger signs listed and each mother was able to identify one form of cause and danger sign or the other.

**Table 2:** Knowledge of causes and danger signs and symptoms of childhood fevers

Variables	Urban households	Rural households	χ2	*P-*value
	(n = 276)	(n = 124)		
	N (%)	N (%)		
**Causes of childhood fevers**				
Malaria	232 (84.0)	124 (100)	22.16	<0.001
Measles	214 (77.5)	100 (80.6)	3.22	0.073
Pneumomia	183 (66.3)	99 (79.8)	7.52	0.006
Diarrhoea	134 (48.5)	75 (60.5)	4.87	0.027
Otitis Media	57 (20.6)	16 (12.9)	3.43	0.064
Meningitis	78 (28.2)	28 (22.6)	1.41	0.234
Recent Immunization	214 (77.5)	97 (78.2)	0.02	0.878
Aware of all listed causes	0 (0)	0 (0)	NA	NA
Don't know	0 (0)	0 (0)	NA	NA
**Danger Signs and Symptoms**				
Convulsion	199 (72.0)	118 (95.2)	27.60	<0.001
Strange breathing	80 (29.0)	60 (48.4)	14.12	<0.001
Confused	27 (9.8)	22 (17.7)	5.03	0.025
Vomiting	42 (15.2)	20 (16.1)	0.05	0.816
Diarrhoea	174 (63.0)	84 (67.7)	0.82	0.364
Anaemia	47 (17.0)	60 (48.4)	42.83	<0.001
Blueness of lip/tongue	17 (6.1)	20 (16.1)	10.11	0.001
Aware of all listed danger signs	0 (0)	0 (0)	NA	NA
Don't know	0 (0)	0 (0)	NA	NA

NA = Not applicable

### Sources of treatment

Table 3 shows that both urban and rural respondents would use self-medication at home as the first line of treatment when a child has fever with urban respondents being more likely to do so (urban 72.5% vs. rural 58.1%). For those who did not recover after the first action, where they sought help for their second action was determined. Thus the private clinics (Urban 49.8% vs. rural) general hospital (Urban 42.9% vs. rural 19.6%), and health centers (Urban 7.3% vs.rural 38%) are used. Overall, the first and second actions taken were statistically significantly different between the urban and rural respondents (*P* = 0.0001 respectively). Paired comparison to detect where the actual differences occurred is shown in table 3.

**Table 3:** First and second actions taken during illness in under-five children

First action	**Urban households**	**Rural households**	**Χ2**	***P*-value**
	**(n = 276)**	**(n = 124)**		
	**N (%)**	**N (%)**		
Drug given at home	200(72.5)	72(58.1)	8.15	0.004
Taken to patent medicine dealer	20 (7.2)	27 (21.8)	.17.41	<0.001
To traditional healer	2 (0.7)	10 (8.1)	-	<0.001*
To health centre	19 (6.9)	3 (2.4)	3.28-	0.070
To hospital	11 (3.9)	0 (0)	-	0.016*
To private clinic	14 (5.1)	1 (0.8)	-	0.027*
Village health worker	0 (0)	10 (8.1)	-	<0.001*
Mean time and standard deviation before first action was taken	1.05±1.67 days	2.32±0.82 days;	-	0.037
**Second action**	Urban	Rural	χ2	*P*-value
	(n = 233)	(n = 92)		
	N (%)	N (%)		
To health centre	17 (7.3)	35 (38.0)	46.40	<0.001
To hospital	100 (42.9)	18 (19.6)	15.55	<0.001
To private clinic	116 (49.8)	39 (42.4)	1.45	0.229
Drugs given at home	-	-	-	
Taken to patent medicine dealer	-	-		
To traditional healer	-	-		
Mean time and standard deviation before second action was taken	2.81±1.06 days	4.5±0.57 days;	-	0.003

* based on Fisher's exact

The time before first action was taken for childhood fevers in urban (1.05±1.67 days with a 95% confidence interval of 0.99 to 1.11 days) was significantly shorter than in rural areas (2.32±0.82 days with a 95% confidence interval of 2.12 to 2.52). This trend was also found in the second action (urban, 2.81±1.06 days with 95% confidence interval of 2.65 to 2.97 vs. rural, 4.5±0.57 days with a 95% confidence interval of 4.1 to 4.9 days). .

### Drugs used in the treatment of childhood fever

As shown in table 4, the main anti malaria drugs taken at home for childhood fevers presumed to be malaria are Chloroquine (44.4% rural vs 48% urban), Coartem (an artemisinin-based combination therapy) (8.3% rural vs 5% urban), and SP (2.8% rural vs 3.5% urban). Aspirin (11.1% rural vs. 6% urban) and Paracetamol (87.5% rural vs 66.6% urban) are the main antipyretics drugs given at home. Other non-specific drugs given are cough syrups, multivitamins and haematinics. The statistical differences in usage between rural and urban respondents is shown in table 4. Rural respondents were more likely to use drugs from many sources than the urban respondents. Both used mostly drugs from the patent medicine dealers, but usage of the leftover drugs and drugs from neighbours were more popular options among the rural respondents than the urban respondents

**Table 4:** Drugs given by mothers as home care and their sources

	Urban households	Rural households	χ2	*P*-Value
	(n = 200)	(n = 72)		
	N (%)	N (%)		
**Drugs**				
Paracetamol	133 (66.5)	63 (87.5)	11.55	<0.001
Aspirin	12 (6.0)	8 (11.1)	176.0	<0.001
Chloroquine	96 (48.0)	32 (44.4)	0.27	0.604
Cough syrup	121 (60.5)	53 (73.6)	3.95	0.047
Native Concotion	6 (3.0)	4 (5.6)	-	0.256*
(sulfadoxine/pyremethamine (SP)	7 (3.5)	2 (2.8)	-	0.769*
Coartem (ACT)	10 (5.0)	6(8.3)	-	0.225*
Multivitamins	72 (36)	56 (77.8)	37.09	<0.001
Blood tonic	31 (15.5)	26 (36.1)	13.58	<0.001
**Sources of drugs**				
Left over from previous hospital visit.	98 (49.0)	58 (80.5)	21.55	<0.001
Bought from patent medicine dealer.	143 (71.5)	68 (94.4)	16.02	<0.001
From friends/Neighbours	9 (4.5)	17 (23.6)	22.37	<0.001
From a traditional healer.	3 (1.5)	5 (6.9)	-	0.032*

* based on Fisher's exact

### Cost of treatment

The mean total cost of treatment, including transport and drugs was 325.5 Naira, ($2.5) with a 95% confidence interval of 270.9 Naira ($2.08) to 380.1 Naira ($2.95) in urban and 220 Naira ($1.69) with a 95% confidence interval of 160.2 Naira ($1.23) to 279.8 Naira ($2.15) in rural area

### Preventive measures used

Table 5 shows that the use of preventive measures against childhood fevers is common in both communities with urban respondents being more likely to use a preventive measure than the rural respondents (76.8% urban vs. 56.4% rural) which was statistically significantly different. The main preventive measures used were insecticide treated and ordinary nets, insecticide aerosols, mosquito coils, pyrimethamine and environmental management.

**Table 5:** Preventive measures used by the mothers

Measures	Urban households	Rural households	χ2	P-value
	(n = 276)	(n = 124)		
	N (%)	N (%)		
Use of Preventive measures	212 (76.8)	70 (56.4)	17.05	<0.001
Type of Preventive measure used	(n = 212)	(n = 70)		
	N (%)	N (%)		
Use of insecticide-treated/ordinary net	93 (43.9)	19 (27.1)	6.15	0.013
Flitting the house with insecticides	24 (11.3)	1 (1.4)	6.37	0.011
Environmental management	80 (37.7)	20 (28.5)	1.93	0.164
Use of Pyrimethamine	57 (26.9)	6 (8.6)	10.17	0.001
Use of mosquito coils	66 (31,1)	31 (44.3)	4.03	0.044

## Discussion

The results of this study offer some insight into the behaviour of urban and rural women in response to childhood fever. Malaria was mentioned by most respondents in both areas as the commonest cause of childhood fevers, a finding that has been noted elsewhere in Nigeria [31]–[33]. In malaria endemic areas, fever is regularly taken as a proxy for malaria, although it may have other causes. In Africa, it has been found that more than 50% of patients identified from their symptoms as cases of malaria only have illnesses attributable to other causes [34], [35].

Recognition of childhood fever and danger signs is more pronounced in the rural mothers. This result could be attributed to the age and experience of most rural mothers who were found to be more elderly than the urban respondents. This is in contrast to the report elsewhere [15].

Most mothers treated their child at home first using available drugs or herbs within the household. Help is sought from the wider community only when the home remedy fails. A similar result was obtained in previous studies [36]–[40]. Surprisingly this practice was observed more in the urban areas. A possible explanation might be that efforts by government and non- governmental organizations to improve home management via information, education and communication may have been concentrated in the urban area at the detriment of the rural populace.

Rural households mostly used the services of informal healthcare providers. This trend has been reported elsewhere in Nigeria [7], [41]. Although the Primary health centers which are supposed to be the first port of call of patients in rural areas were not used significantly in their first action, its use increased in the second action significantly. This might be a reflection of the confidence they have in the health centers as a source of care for complicated cases and also perceived them to offer better healthcare than the patent medicine dealers which they used more in their first action. The fact that they did not use the health centers significantly in the first action might be due to their perceived high cost of treatment or that they were not easily accessible geographically. The patent medicine dealers have been known to be both financially and geographically accessible to rural dwellers [42], [43].

The use of formal healthcare services by the urban respondents could reflect the availability of these formal healthcare services in the urban setting. Other determinants could be the difference in educational level and occupational status of the mothers in both areas with the urban mothers being more educated and having higher occupational status than the rural mothers. In Kenya, despite marked differences between the rural and urban areas in population structures and access to treatment providers, rural and urban mothers treatment seeking pattern in relation to childhood fevers were similar [1], although, studies have shown that preventive and curative health-seeking behaviors for children are clearly better in urban than rural areas [44].

The commonest drug used by both rural and urban residents in treatment of childhood fevers was paracetamol. A common phenomenon is that patients and medicine sellers in Nigeria appear to confuse analgesics/antipyretics with antimalarials [45]. . Hence, there is need to educate mothers on the appropriate dosage of paracetamol to be given. Rural mothers are also more likely to use chloroquine, Nivaquine, sulfadoxine/pyremethamine (SP) and multivitamins than the urban mothers. This could be attributed to their less frequent use of modern health facilities like private, general hospitals and hence they relied more on the drugs bought from patent medicine dealers. The cost and availability of drugs may have also contributed to this as these drugs are known to be cheaper and readily available than other anti-malarial drugs like Halofantrine and artemesinin-based combination therapy (ACTs). However the use of an ACT in both areas despite their multi-dosage/multi-day regimens and high cost was surprising. This might be an indication that the community members are beginning to tolerate ACT products as this has now been officially introduced in Nigeria as first line antimalaria drugs. It will therefore be worthwhile to investigate the feasibility of deploying ACTs in the community either within the home management programs or through the private sectors like the patent medicine dealers.

Furthermore, drugs given by mothers in both areas were mainly obtained from patent medicine dealers. It has been noted elsewhere in Nigeria that these PMS are usually the first choice in health care and a recognized primary source of orthodox drugs for both rural ad urban populations, especially the poor [7], [32], [46]. Ordinarily the patent medicines should be sold in their original packs. Over-the- counter (OTC) drugs are the only drugs authorized to be sold by the vendors, but they generally sell all types of drugs as determined by their financial capability [47] and these range from paracetamol and chloroquine in large tins to antibiotic, psychotropic drugs, narcotics, toxoids, and antihypertensives, that are outside the scope of their license [48], [49]. In addition, they obtain their drug supplies through both formal and informal channels including large retail and wholesale pharmacies in major cities, direct from pharmaceutical companies, and through visiting company representatives [49]. There are reports that these drugs may be ineffective, counterfeit or expired [48]. This undermines therapeutic efficacy and may promote the emergence and further spread of drug-resistance.

The rural mothers are also more likely to get drugs from their friends/neighbours than the urban mothers. This could be due to the homogenous nature of the rural residents with regards to culture while the urban dwellers are heterogeneous coming from different cultural backgrounds and places. Since mothers are already sourcing their treatment from the patent medicine dealers, and treating their children at home, the training of both caregivers and the patent medicine vendors is advocated in order to meet the objectives of home management and early and appropriate treatment and referral of severe cases.

Some of these patients may have gotten better after taking drugs from either PMD, Village Health Workers or Traditional healers. Hence the effectiveness of self medication and non medical prescribing needs to be researched to gain an insight what may be playing out there.

The time before first action was taken in urban was significantly shorter than in rural areas. One reason for this could be nearness of health facilities in urban areas. The rural people are also predominantly poor and may not be able to pay their fees thus delaying action even though the cost of treatment in rural areas is less than that in urban areas. Also if the fever occurred during the farming season, the rural mothers may not have enough time to take their children to health facilities on time as they will be busy in their farms. Awareness creation on the need to seek help as soon as illness starts especially in rural areas will help to improve the situation.

Also, construction of more PHC centers in rural areas will likely improve the physical accessibility of health facilities.. It has been noted that the geographical proximity of services to peoples' homes is one of the most important factors that affect utilisation of health services, particularly in rural areas of developing countries [50]–[52]. As distance increases, the level of utilisation decreases and vice versa, and that hence people who live far away from services suffer a greater disadvantage regarding the use of services if they are also poorer and transport is expensive [50]. Subsidized payment or targeted exemption system especially in the rural areas will also improve their health seeking behaviour. However, strengthening access to health systems as a whole in rural areas is an important feature in improving health seeking behaviour amongst both urban and rural dwellers.

The lower costs incurred by the rural residents may actually reflect the fact that they received less healthcare than the urban. It may also reflect the fact that they used the lower level health care providers like the patent medicine dealers more which is cheaper. It is also possible that they may not have been able to pay for all of the drugs prescribed them. The high frequencies of treatment without accurate diagnosis and of the patronage of low-level providers already mean that there is much irrational and/or ineffective treatment of fevers and malaria, which in turn leads to high and unnecessary individual and societal costs [53], [54].

The urban residents are more likely to use a preventive measure. This urban-rural difference may be expected considering the fact that urban population is more exposed to advocacy and regular information from the media on malaria preventive measures. The generally lower usage of preventive measures against malaria in the rural area except for mosquito coils could be attributed to the better socio-economic background of the urban respondents. Non-availability and cost may also be responsible for the low usage of ITN in the rural area. However, a qualitative sturdy will be necessary to discern the reason for this pattern of use.

In Nigeria the current national key strategies to roll back malaria include that at least 60% of those affected by malaria have rapid access to, and are able to correctly use, affordable and appropriate treatment within 24 hours of the onset of symptoms and that at least 60% of those at risk from malaria, particularly children under five years and pregnant women, benefit from the most suitable combination of protective personal and community measures such as insecticide-treated nets and other accessible, affordable interventions to prevent infection and suffering; and that at least 60% of all pregnant women at risk from malaria, especially those in their first pregnancies, have access to chemoprophylaxis or intermittent preventive treatment.[55]. The study indicates that this is still far from be achieved as there are still delays in accessing appropriate treatment and use of appropriate preventive measures. By the end of 2005, only 34.6% had treatment within 24hours of onset of Symptoms in Nigeria, while.11.9% had aappropriate treatment [56]. As regards malaria prevention in pregnancy, 34% had chemoprophylaxis in the urban region while in the rural area, only 15% had chemoprophylaxis.

### Conclusions

Urban and rural mothers are aware that malaria is the major cause of childhood fevers, but differences exist in their knowledge of signs and symptoms of childhood fevers, ability to recognize danger signs and symptoms, preventive measures, type of health facility used and responses to childhood fever which may be important for targeting for National malaria control programs for maximum effects. The relative impacts of targeted control and random control have been demonstrated [57]. As has been shown, because of the underlying differences between rural and urban areas [15]–[17], [31], fever/malaria prevention and control measures may have to be modified in both environments. Information education and communication materials on signs and symptoms of childhood fevers and possible actions to be taken by mothers should be made fully available to mothers and caretakers in both urban and rural areas. Emphasis should be laid on appropriate home treatment and preventive measures of childhood fevers during health education sessions given to mothers during their antenatal, postnatal and immunization visits especially to the rural mothers.

Available formal and non-formal health care options include home management, patent medicine dealers and health facilities in both urban and rural areas. They all have great potentials for successful implementation of malaria control programmes in both areas and should be explored by policy makers and programme managers if Malaria-related Millennium Development Goals (MDG 4) is to be achieved.
